# An algorithm for cardiac disease detection based on the magnetic resonance imaging

**DOI:** 10.1038/s41598-025-88567-3

**Published:** 2025-02-03

**Authors:** Heng Li, Qingni Yuan, Yi Wang, Pengju Qu, Chunhui Jiang, Hu Kuang

**Affiliations:** 1 Key Laboratory of Advanced Manufacturing Technology of the Ministry of Education, Guizhou University, Guiyang, 550025 China; 2 The First People’s Hospital of Guiyang, Guiyang, 550002 China

**Keywords:** Object detection, Cardiac MRI medical images, Cardiac diseases, Spatial pyramid pooling, Joint attention mechanism, Bounding box regression loss, Magnetic resonance imaging, Computer science, Diseases, Medical research

## Abstract

In experiments to detect heart disease on cardiac magnetic resonance imaging (MRI) medical images, existing object detection models face several challenges including low accuracy and unreliable detection results. To tackle these issues, this article proposes an innovative method for Object Detection in cardiac MRI medical images called SA-YOLO. This method is based on the YOLOv8 model but introduces several key modifications. Firstly, the standard Spatial Pyramid Pooling Fast module is replaced with a Multi-Channel Spatial Pyramid Pooling module. Secondly, an attention mechanism combining the ideas of Squeeze-Excitation and Coordinate Attention designed, and integrated into the Neck part of the baseline model. Subsequently, the bounding box regression loss function CIoU loss of the model was replaced with the iSD-IoU loss that combines shape loss and distance loss. Finally, comparative experiments were conducted on the Automated Cardiac Diagnosis Challenge cardiac MRI image dataset where it was found that SA-YOLOv8 achieved better results in detecting cardiac pathologies, and improvement of 7.4% in mAP0.5 value and 5.1% in mAP0.5-0.95 value compared to the baseline model.

## Introduction

In recent decades, cardiovascular diseases have emerged as a prominent cause of impaired health and reduced lifespan in humans. According to data released by the World Heart Federation (WHF), cardiovascular diseases consistently rank as the primary global cause of mortality, affecting over 500 million individuals worldwide. In 2021 alone, approximately 20.5 million people succumbed to cardiovascular diseases, accounting for an astonishing one-third of total deaths globally^1^.

Heart disease is a significant component of cardiovascular disease. In recent years, MRI technology has been widely utilized in the cardiac medicine field. However, analyzing cardiac MRI images requires specialized knowledge and experience from cardiologists due to the unique characteristics of the heart’s complex structure and variations in size and shape among individual patients’ hearts. As a result, diagnosing cardiac pathology is a knowledge-intensive task that demands significant investments of resources to train qualified cardiac physicians. This process is both expensive and time-consuming. With the increasing number of patients each year in this field alone, relying solely on training new physicians to address the shortage of medical resources becomes impractical, thus necessitating the exploration of alternative solutions.

Thanks to the application of deep learning techniques in machine vision tasks, significant advances have also been made in Image Segmentation^2,3^ and Object Detection (OD) related technologies. OD models effectively learn pathological features from medical images, enabling accurate and rapid detection and identification of diseased tissues or organs. Significant research advancements have been made in various medical fields, including segmentation or detection of brain diseases^4,5^, cell detection^6^, breast lesion identification^7,8^, skin disease detection^9^, lung disease recognition^10^, and bone fracture detection^11^, and the same is true of research into the diagnosis of heart disease. For example: Song, B^12^ conducted research on the automatic segmentation of the biventricular heart and classification of heart diseases using cardiac MRI medical images, achieving satisfactory results in classification and segmentation. Lin, Y^13^ researched the auxiliary diagnosis and treatment of congenital heart disease using deep learning, proposing a lesion detection network based on refined SSD, which achieved the detection of congenital heart disease from cardiac ultrasound images. Qiao, S. et al.^14^ proposed a residual learning diagnostic system RLDS based on convolutional neural networks (CNNs) for congenital heart disease in fetuses. They conducted experiments on a self-made dataset of fetal echocardiography, where the RLDS model achieved detection accuracy and recall rates of 93% for fetal coronary heart disease. Jothiaruna, N. et al.^15^ proposed a method using deep neural networks to detect heart diseases, achieving a classification accuracy of 95.88% mAP on 12-lead electrocardiogram images. Wu, B. et al.^16^ proposed a machine learning-based two-dimensional ultrasound image fetal cardiac defect feature extraction/recognition model, achieving a maximum classification accuracy of 87.35%. Doppala, B. et al.^17^ introduced a lightweight Tiny 2D-CNN model for detecting cardiac enlargement in cardiac CT images, with a classification accuracy of 96.32%. Paul, V. V. et al.^18^ through the investigation and analysis of various cardiovascular disease prediction methods, the advantages and disadvantages of different algorithms have been revealed, providing valuable insights for future research. Wang, Z.et al.^19^ gave a comprehensive overview of 3D vision-based cardiovascular imaging, explored its future applications in cardiovascular image analysis, and reviewed the most advanced 3D vision methods for vascular tasks, providing effective assistance for heart disease-related research.

Generally, deep learning-based OD algorithms have made significant progress in recent years, and there is still a lot of untapped potential in cardiac medicine. The current OD model faces challenges and limitations in cardiac MRI medical image detection. For example, the annotation of cardiac MRI images requires the participation of medical experts, which is costly, time-consuming, and laborious. The accuracy and consistency of annotation may also affect the training effect of the model. Cardiac MRI images may come from different modalities (such as different contrast images or different imaging slices), and the differences between these modalities pose great challenges to target detection. Some cardiac MRI images have a slightly lower resolution, resulting in some lesions or structures that cannot be accurately detected or organ detection confusion problems. High-precision target detection models often require a lot of computing resources, are not easy to deploy, and have insufficient generalization capabilities. Because of these problems, most models performed poorly when the author’s team used cardiac MRI image datasets for model training. Additionally, researchers predominantly rely on echocardiograms^14,20,21^, CT images^17,22,23^, and electrocardiograms^15,24,25^ as their primary data sources, with relatively fewer studies focusing on cardiac MRI images.

To resolve these concerns, the article proposes a novel OD model for diagnosing cardiac pathology called SA-YOLO. This model is built upon the YOLOv8^26^ network architecture and incorporates an innovative Multi-Channel Spatial Pyramid Pooling (SPPMC) module and attention mechanism (UECA) module that unites the ideas of squeeze-excitation and coordinating attention while optimizing the Bounding Box Regression (BBR) loss function. In order to validate the effectiveness and advantages of our proposed method, extensive ablation, and comparative experiments were performed using the publicly available Automatic Cardiac Diagnosis Challenge (ACDC) cardiac MRI image data^27^. The experimental results demonstrate that our improvement strategies significantly enhance the performance of both the baseline model and other models when integrated with them. Furthermore, in comparative experiments with other cutting-edge OD models, our SA-YOLO model achieves superior detection and recognition results compared to the baseline model, exhibiting an improvement of 7.4% in mAP0.5 metric and 5.1% in mAP0.5-0.95 metric.

**Fig. 1:**
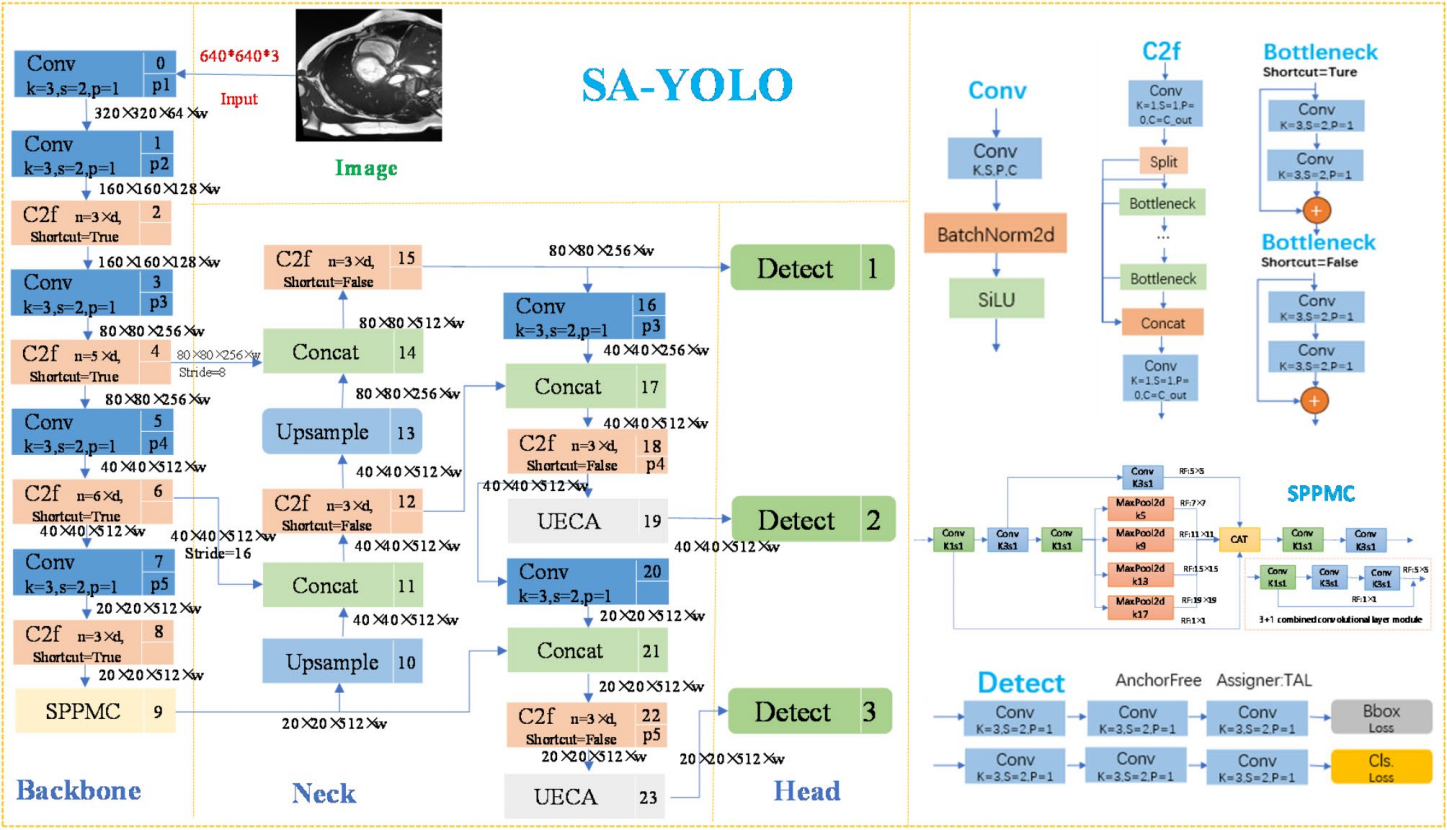
Network structure diagram of the SA-YOLO model.

The findings of this paper are outlined below:

- Designed a Multi-Channel Spatial Pyramid Pooling Network Module (SPPMC). This module consists of parallelized max-pooling layers and convolutional layers with varying sampling rates, effectively enlarging the receptive fields within the network. Such design enables the extraction of comprehensive feature information from input feature maps, encompassing both fine-grained and coarse features. Moreover, this module adopts a strategy of separately processing and subsequently integrating extracted global or local feature information, thereby reducing interference and redundancy between global and local information. This enhances the model’s capacity to accurately represent input data features while improving its efficiency in handling complex data.
- Designed the UECA module to integrate the ideas of the Squeeze-and-Excitation Attention Network (SE)^28^ and the Coordinate Attention Network (CA)^29^. This module synergistically combines the squeeze-and-excitation submodule and the coordinate information embedding submodule in a weighted parallel manner, enabling comprehensive attention consideration across both channel and spatial dimensions. This design effectively enhances the network’s capacity to capture contextual information of targets and expand its perception range. Furthermore, this mechanism dynamically adjusts spatial and channel weights through learning, reinforcing the representation of crucial features while suppressing irrelevant feature interference.
- The SPPMC and UECA modules have been seamlessly integrated into the YOLOv8 architecture. Additionally, we propose optimizations to the BBR loss function to address challenges associated with unreliable cardiac pathology detection and enhance model robustness in cardiac MRI medical imaging. Experimental results on the ACDC dataset demonstrate that our SA-YOLO model surpasses both the baseline model and other cutting-edge OD models.

## Related works

In the context of cardiac pathology target detection, the key to improving the performance of target detection models and helping the network to better detect and classify cardiac pathology in MRI medical images is how to extract richer multilevel features, how to enhance and fuse the extracted features, how to optimize the training process, and how to improve the localization accuracy of the model. This paper will provide solutions to the above problems by studying spatial pyramid pooling networks, attention mechanisms, and loss function optimization.

### Spatial pyramid pooling

Spatial Pyramid Pooling (SPP) was originally proposed by Kaiming He et al.^30^. The main role of the SPP module in backbone networks is to solve the limitations brought by different sizes of image inputs in neural networks, to help the network capture richer, more comprehensive, and high-quality feature information, to provide the network with powerful contextual information, and to enhance the robustness of the model without introducing significant computational costs, etc., so the design of the SPP module structure is fundamental. Subsequent researchers have proposed a variety of variants on this basis, such as the standard Spatial Pyramid Pooling Fast (SPPF), SPPCSPC, SPPELAN, etc.

The SPPF reduces the computational cost and improves the computational efficiency through the convolutional sharing operation, but the modification is relatively limited; The SPPCSPC combines the advantages of the SPP and Cross Stage Partial (CSP) structures and enhances feature expression capability, but the computational cost is increased, and the feature details are easily lost after the iteration of multiple convolutional layers. The SPPELAN combines the ideas of SPP and Efficient Layer Aggregation Network (ELAN), which further increases the capability of feature fusion and feature expression, but the complexity of the model structure is increased.

### Attention mechanism

The attention mechanism plays an important role in feature enhancement and fusion, after the introduction of the attention mechanism in the Neck part of the target detection model, the network can dynamically adjust the feature weights according to the input feature maps, inhibit the perturbation of non-essential features, and contribute to the enhancement of the characterization ability of important features. In addition, the introduction of the attention mechanism can effectively promote multi-scale feature fusion, enabling the model to simultaneously utilize high-level semantic information, and low-level detail information plays a positive role in model performance enhancement. Commonly used attention mechanisms include SE, CA, Convolutional Block Attention Module (CBAM)^31^, etc.

The SE attention mechanism demonstrates a straightforward structure and convenient usage, enabling neural network models to selectively emphasize valuable features by leveraging global information while suppressing fewer valuable ones. However, it solely focuses on channel-wise attention without considering spatial attention, thus possessing certain limitations. The CA attention mechanism incorporates location information into channel attention and expands the network’s attention span. Additionally, it facilitates adaptive selection and weighting of channel features, enhancing the network’s ability to focus on crucial channel information. However, the CA attention mechanism primarily emphasizes spatial relationships between pixels, which poses certain limitations in handling long-range dependency relationships. The CBAM attention mechanism processes feature maps sequentially through two submodules, channel attention, and spatial attention, which take into account the feature enhancement in both channel and spatial dimensions, and the network can pay more attention to the important features, which improves the model’s performance ability. However, its mode of computing channels attention first and then spatial attention limits the flexibility of the attention mechanism to some extent.

### Loss function optimization

A good loss function design can help the target detection model to improve the localization accuracy, optimize the training process, accelerate the model convergence and so on. In recent years, BBR loss functions have made very rapid development in target detection, such as DIoU^32^, CIoU^32^, SIoU^33^, Shape-IoU^34^, Inner-IoU^35^, etc. DIoU introduces a centroid distance penalty term on the basis of IoU^36^, which takes into account the influence of the overlapping area of the Bounding (BD) box and the distance between the centroids of the BD box. CIOU is improved from DIoU and introduces a new shape loss, which takes into account the transverse area between the Anchor box and the Ground truth (GT) box. CIoU is improved from DIoU by introducing a new shape loss, taking into account the difference in aspect ratios between the Anchor box and the GT box, and optimizing the location and shape of the BD box. SIoU is optimized in terms of the location, size, aspect ratio, and angle of the BD box, and the introduction of the angle penalty makes it shine in the task of detecting the tilted box. Shape-IoU focuses on the influence of the shape and scale factor of the BD box on the regression results. Shape-IoU pays more attention to the influence of the shape and scale factor of the BD box on the regression results to make the bounding regression more accurate. Inner-IoU calculates the IoU loss by designing an auxiliary BD box and accelerates the convergence by using different sizes of the BD box for the high IoU and low IoU samples, respectively.

Based on the above research, this paper proposes the SPPMC module, the UECA module, and the iSD-IoU loss function, respectively, to enhance the detection accuracy and reliability of the target detection model. The SPPMC module connects the backbone network and the neck network and has further enhanced the ability to extract the multi-scale features, improves the richness of feature information, and at the same time, the network’s adaptive ability has been improved, and the stable propagation of gradient is maintained, and the loss of feature information is reduced, etc. The design of the UECA module makes up for the lack of CA and SE attention mechanism, while considering the attention of spatial dimension and channel dimension, accessing the neck network, which further enhances the characterization ability of important features, and promotes the high-quality fusion of feature information. iSD-IoU loss function is designed by introducing the idea of an auxiliary bounding box to calculate IoU and combining the distance loss, and shape loss effect to further enhance the training of the model optimization model, accelerate the convergence speed, and improve the positioning accuracy.

## Methods

### Improved SA-YOLO network model

After incorporating the SPPMC module, UECA module, and BBR loss function iSD-IoU loss into the network architecture of YOLOv8, the resulting SA-YOLO model exhibited exceptional performance in complex cardiac pathology detection tasks. This model effectively identifies normal human cardiac structures as well as previous myocardial infarction cases, hypertrophic cardiomyopathy conditions, dilated cardiomyopathy instances, and abnormal right ventricle abnormalities. This innovative approach offers a fresh perspective for advancing research and development in cardiac pathological detection methods. Its primary objective is to overcome challenges associated with detecting cardiac pathologies in medical images obtained from cardiac MRI scans, thereby fostering scientific advancements in medical imaging technology while enhancing accuracy and efficiency in diagnosing and treating heart diseases by healthcare professionals.

The network architecture of the SA-YOLO model proposed in this paper is depicted in Fig. 1. Taking a 640 × 640-pixel input image as an example, it undergoes feature extraction through a backbone network, resulting in three effective feature layers sized 80 × 80, 40 × 40, and 20 × 20 respectively. Then, the network will input the above extracted multi-scale features into the neck for feature enhancement and fusion so that it can meet the requirements of the target detection task. Finally, the Head part of the model will utilize these fused and enhanced feature information for cardiology detection and classification, and generate the final detection results.

It is worth noting that the design of the SPPMC module’s adding position as the last layer of the backbone part, can help the network better process targets of different scales, extract feature information, enhance the richness of network features, improve the robustness of detection, optimize the computational efficiency, and thus improve the detection performance of the entire model. In the cardiac MRI image data, the heart organ occupies large pixels, which can be used as medium and large-scale targets. The second detection head of YOLOv8 accesses the feature map at the middle level of the network, which is responsible for detecting medium-scale objects; and the third detection head accesses the feature map at the deeper level of the network, which is responsible for detecting objects at large iso-scales. Thus, in this paper, we add the UECA module before the second and third detection heads of the model and enhance the network’s ability to focus on its key features and contextual information processing, in order to improve the quality of the feature representations, which in turn significantly improves the overall performance of the model.

### The multi-channel spatial pyramid pooling module

In cardiac MRI images, due to the specificity of heart size, the scale differences of various cardiac pathology features, and the effect of image resolution, this can pose a great challenge to the model’s multi-scale information extraction and fusion capabilities. Traditional convolutional neural networks are usually more sensitive to scale differences and difficult to handle multi-scale features simultaneously, and the traditional Spatial Pyramid Pooling module utilizes a fixed-size pooling layer, which may limit the size of the receptive field, fail to effectively capture comprehensive feature information, and easily lose the details during the convolutional iteration process. To address this problem, this paper combines the characteristics of cardiac MRI medical images, this paper designs a SPPMC module for cardiac pathology detection tasks, which enables the model to simultaneously process abnormalities at more scales, helps the network model to improve the ability to capture and fuse multi-scale feature information, while reducing the loss of useful information, and thus improves the ability to detect a wide range of cardiac pathologies. The structure diagram is depicted in Fig. 1.

Inspired by the SPPCSPC module^37^, the design of SPPMC incorporates four max-pooling layers with kernel sizes increases in the order of 5, 9, 13, and 17, taking into account the feature abstraction level, multi-scale information requirements, and task requirements. This design strategy not only helps the network capture a wider range of context information but also helps the network obtain an appropriate amount of shallow features and deeper features simultaneously, enhancing the diversity and completeness of feature representation. The pooling of multiple scales can reduce information redundancy to a certain extent, and ensure that some features that are more significant at a certain scale will not be ignored; When processing data, the network can easily deal with all kinds of data and extract the most representative features, which increases the flexibility and adaptability of the network. Furthermore, the design of the pooling kernel size in the max pooling layer adheres to an arithmetic progression, enabling the network to produce a uniformly varying receptive field across both width and height dimensions, and further enhancing the coverage range of the receptive field, avoiding the risk of overfitting caused by a single scale change and the situation of uneven or discontinuous feature extraction, ensuring that the network’s perception ability of input data at all scales is balanced. It is helpful for the network to capture more sensitive features at different scales to obtain higher-quality feature information. In addition, the jump connection of the parallel residual network structure is conducive to the stability of gradient propagation, alleviates the situation of feature loss, and provides the network with richer and more diversified features. The smooth and non-monotonic Sigmoid Linear Unit (SILU) function^38^ is chosen as the activation function in this module to help the model always maintain more effective information propagation and gradient updating, etc. during the practicing process.

The receptive field in convolutional neural networks is defined as the region is located in the feature map of each layer that corresponds to pixels on the original input image. Equations (1) and (2) illustrate the calculation method for determining the receptive field in the network. Here, *R* represents the overall receptive field, $\:{R}_{n}$ represents the receptive field of layer n, $\:{k}_{n}$ represents the convolution kernel of layer n, and $\:{s}_{i}$ denotes the step size of Layer i. According to Eqs. (1) and (2), the SPPMC module can generate a relatively uniform variation of the receptive fields before feature fusion, whose dimensions are 1 × 1, 5 × 5, 7 × 7, 11 × 11, 15 × 15 and 19 × 19, respectively.

1 $$R_{0} = 1;R_{1} = k_{1}$$

2 $$\:{R}_{n}={R}_{n-1}+\left({k}_{n}-1\right)*\prod\:_{i=0}^{n-1}{s}_{i}\:\:n\ge\:2$$

### Joint attention mechanisms incorporating SE and CA

In cardiac MRI images, the image composition is complex, often mixed with other organs and tissues, and the background noise is large, which poses a challenge to the extraction and fusion of key cardiac lesion feature information, and there are some limitations on the focus range or flexibility of some traditional attention mechanisms. To address this problem, this paper draws on SE and CA attention mechanisms to design the UECA attention mechanism module, which take into account both spatial and channel dimensions, and improve the global context perception ability, which can adaptively strengthen the key features in the channel dimension, and also improves the ability of the model to pay attention to the key features in the spatial dimension, to enable the model to face the complex cardiac lesion scenarios. In addition, the parallel computation of channel attention and spatial attention not only increases the flexibility of the attention mechanism, but also allows the model to optimize in different dimensions at the same time, which improves the feature selection and expression ability.

**Fig. 2:**
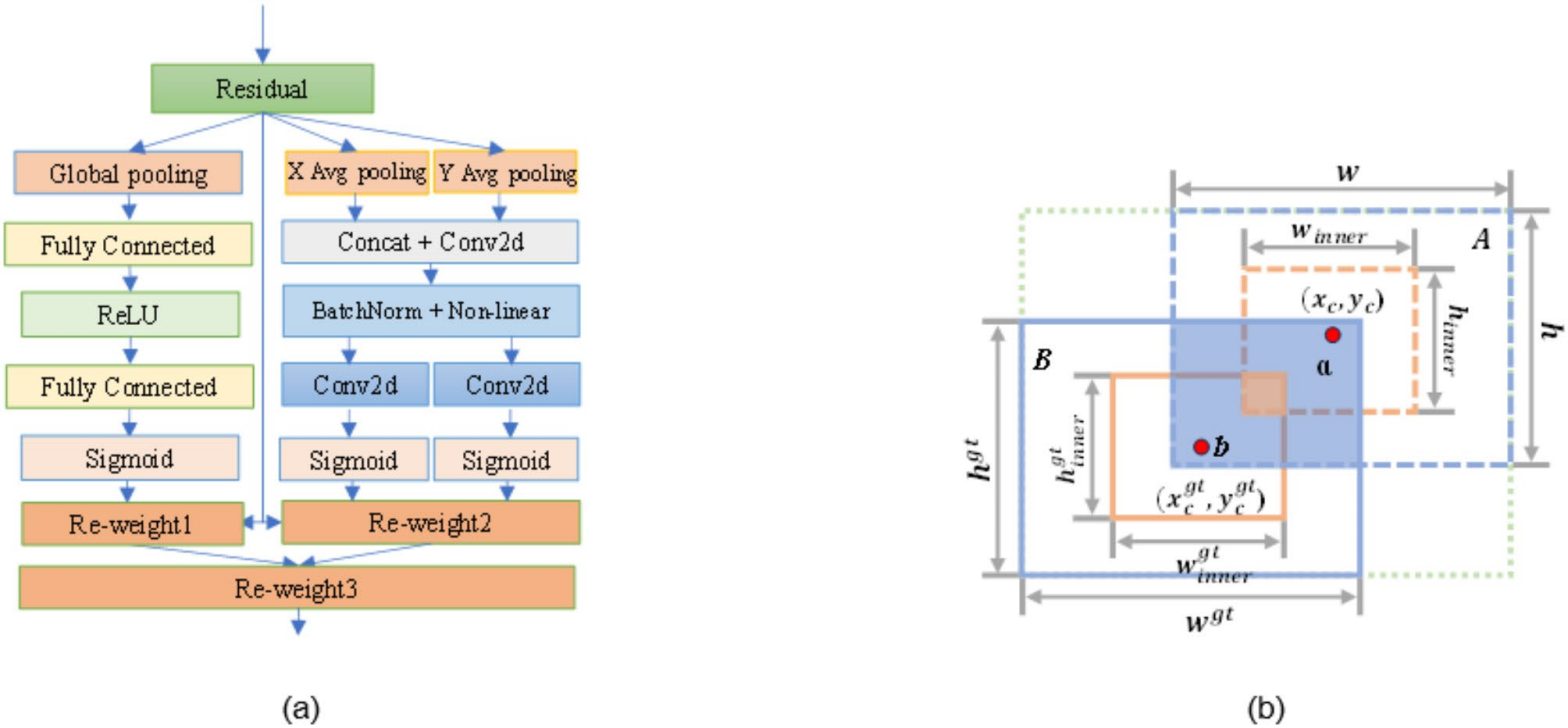
The structure diagram of modules: (**a**)The structure of the UECA module. (**b**) Descriptive diagram of iSD-IoU.

As depicted in Fig. 2(a). The UECA module consists of two parallel-weighted submodules capable of processing feature information across different dimensions, significantly enhancing model performance. The UECA module functions as an independent computational unit, transforming the input tensor **X** into the output tensor **Y**, as demonstrated in the following equation:

3 $$\:\varvec{Y}=\varvec{X}\text{*}{(\varvec{L}}_{1}+{\varvec{L}}_{2})$$

4 $$\:{\varvec{L}}_{1}=\varvec{X}\text{*}{\varvec{s}}_{1};\:{\varvec{L}}_{2}=\varvec{X}\text{*}{\varvec{s}}_{2}$$

5 $$\:{z}_{C}=\frac{1}{\text{H}\times\:\text{W}}\sum\:_{i=1}^{H}\sum\:_{j=1}^{W}{x}_{C}(i,j){,.z}_{C}\epsilon\varvec{z}$$

6 $$\:{\varvec{s}}_{1}=\rho\:\left({\varvec{M}}_{1}\partial\:\left({\varvec{M}}_{2}\varvec{z}\right)\right),\:{\:\:\:\varvec{M}}_{1}\in\:{\mathbb{R}}^{\frac{C}{r}\times\:C};\:{\varvec{M}}_{2}\in\:{\mathbb{R}}^{C\times\:\frac{C}{r}}$$

7 $$\:{z}_{C}^{h}\left(h\right)=\frac{1}{\text{W}}\sum\:_{0\le\:i\le\:W}{x}_{C}\left(h,i\right),\:{z}_{C}^{h}\left(h\right)\epsilon{\varvec{z}}^{h}$$

8 $$\:{z}_{C}^{w}\left(w\right)=\frac{1}{\text{H}}\sum\:_{0\le\:j\le\:H}{x}_{C}\left(j,w\right),\dots\:{z}_{C}^{w}\left(w\right)\epsilon{\varvec{z}}^{w}$$

9 $$\:\varvec{v}=\partial\:{(F}_{1}\left(\left[{\varvec{z}}^{h},{\varvec{z}}^{w}\right]\right))$$

10 $$\:{\varvec{g}}^{h}={\rho\:(F}_{h}\left({\varvec{v}}^{h}\right));\:\:{\varvec{g}}^{w}={\rho\:(F}_{w}({\varvec{v}}^{w}\left)\right)$$

11 $$\:{\varvec{s}}_{2}={\varvec{g}}^{h}\text{*}{\varvec{g}}^{w}$$

where $\:\varvec{X}=[{x}_{1},{x}_{2},\dots\:\:,{x}_{C}]$ are elements of $\:{\mathbb{R}}^{{C}^{{\prime\:}}\times\:{H}^{{\prime\:}}\times\:{W}^{{\prime\:}}}$,$\:\varvec{Y}=[{y}_{1},{y}_{2},\dots\:\:,{y}_{C}]$ are elements of $\:{\mathbb{R}}^{C\times\:H\times\:W}$; $\:{\varvec{L}}_{1}=[{l}_{1}^{1},{l}_{1}^{2},\dots\:\:,{l}_{1}^{c}]$ are elements of $\:{\mathbb{R}}^{{C}^{{\prime\:}{\prime\:}}\times\:{H}^{{\prime\:}{\prime\:}}\times\:{W}^{{\prime\:}{\prime\:}}}$and $\:{\varvec{L}}_{2}=[{l}_{2}^{1},{l}_{2}^{2},\dots\:\:,{l}_{2}^{c}]$ are elements of $\:{\mathbb{R}}^{{C}^{{\prime\:}{\prime\:}{\prime\:}}\times\:{H}^{{\prime\:}{\prime\:}{\prime\:}}\times\:{W}^{{\prime\:}{\prime\:}{\prime\:}}}$represent the outputs of the left and right submodules, respectively; $\:\varvec{z}\in\:{\mathbb{R}}^{C}$ denotes the result after a series of operations over the spatial dimensions; $z_{C} \epsilon \varvec{z}$ represents the output of the C^th^ channel. $\:{\varvec{s}}_{1}$ indicates the output of the left submodule after capturing channel dependencies; $\:\rho\:$ is the Sigmoid function; $\:{\varvec{M}}_{1}$ and $\:{\varvec{M}}_{2}$ are the parameters for linear transformations; *r* is the reduction ratio; $\:\partial\:$ represents the ReLU function; $\:\varvec{v}$ denotes the feature map obtained after convolution processing; $\:{\varvec{v}}^{h}$ and $\:{\varvec{v}}^{w}$ are the decompositions of $\:\varvec{v}$ in the spatial dimensions *H* and *W*, respectively; $\:{\varvec{g}}^{h}$ and $\:{\varvec{g}}^{w}$are the results of applying convolution operations to $\:{\varvec{v}}^{h}$ and $\:{\varvec{v}}^{w}$, respectively; **s**_2_ represents the product of $\:{\varvec{g}}^{h}$ and $\:{\varvec{g}}^{w}$.

The UECA module has a simple structure and is very suitable for integration into various classic mobile networks. It enables the network to capture richer contextual information and better focus on key features by integrating dimensions such as channel, space, and global information, thereby promoting feature fusion. Through feature extraction in different spatial dimensions, global context information weighting, and global average pooling operations, the network’s ability to capture contextual information can be effectively improved. The network not only captures contextual information in different spatial directions and provides a finer-grained spatial position relationship, but also summarizes the global information of the entire feature map, improving the network’s channel feature expression ability in the global scope. In addition, the UECA module can dynamically adjust the focus between the global and local by extracting feature information in the horizontal and vertical directions, directly expanding the network’s perception range in the spatial dimension and improving its ability to understand features of different scales and positions. By dynamically adjusting channel weights, the network can optimize channel features from a global perspective, and indirectly expand the perception range and suppress irrelevant features, so that the network can more effectively focus on basic cardiac pathological features.

### Optimization of loss function

When dealing with complex cardiac MRI images, the traditional IoU loss function will have problems such as failing to provide accurate localization due to problems such as fuzzy target boundaries, limited ability to optimize the model training process, and failure to quickly help the model converge. To address these problems, this paper designs the iSD-IoU loss function. As shown in Fig. 2(b), the iSD-IoU loss employs the intersection over union of auxiliary boxes ($\:{IoU}^{in}$) and incorporates both distance loss (Δ) and shape loss (Ω) into the BBR loss function, thereby facilitating faster convergence of the bounding boxes and optimizing the overall model training. The introduction of an auxiliary bounding box model allows for a more comprehensive assessment of overlap, position, and shape alignment between predicted (PD) boxes and GT boxes, enhancing detection accuracy. Additionally, incorporating a distance loss model enables simultaneous optimization of both overlap degree and positional accuracy, bringing the center points of PD and GT boxes closer together to improve localization precision. Furthermore, integrating a shape loss model optimizes aspect ratio concurrently, ensuring that the PD box’s shape aligns more closely with the true contour of the target, thus further enhancing localization accuracy. The iSD-IoU loss is defined as follows:

12 $$\:{L}_{iSD-IoU}={1-IoU}^{in}+\varDelta\:+{\Omega\:}$$

The calculation of the $$\:{IoU}^{in}$$ is presented in Eq. (19), where *b* represents the centroid coordinates $$\:({x}_{c}^{gt},$$$$\:{y}_{c}^{gt}$$) of both the GT box and its inner GT box. The upper left and lower right corners of the inner GT frame are denoted by coordinates ($$\:{b}_{l},\:{b}_{t}$$) and ($$\:{b}_{r},$$$$\:{b}_{b}$$), respectively. Similarly, a denotes the centroid coordinates $$\:({x}_{c},\:{y}_{c}$$) of both the PD box and its inner PD box. The upper left and lower right corners of the inner PD box are represented as ($$\:{a}_{l}$$, $$\:{a}_{t}$$) and ($$\:{a}_{r}$$, $$\:{a}_{b}$$). Herein, *r* signifies a scaling factor that relates to target sizes within the dataset ranging from 0.5 to 1.5; *w* and *h* represent width and height values for the PD box, respectively, while $$\:{w}^{gt}$$ and $$\:{h}^{gt}$$ correspondingly denote width and height values for the GT box; $$\:in\_inter$$ refers to intersection area between the inner GT box and inner PD box; finally, $$\:in\_union$$ indicates union area between these two boxes.

13 $$\:{b}_{l}={x}_{c}^{gt}-\frac{{w}^{gt}\text{*}r}{2},\:{b}_{r}={x}_{c}^{gt}+\frac{{w}^{gt}\text{*}\text{r}}{2}$$

14 $$\:{b}_{t}={y}_{c}^{gt}-\frac{{h}^{gt}\text{*}\text{r}}{2},\:{b}_{b}={y}_{c}^{gt}+\frac{{h}^{gt}\text{*}\text{r}}{2}$$

15 $$\:{a}_{l}={x}_{c}-\frac{w\text{*}\text{r}}{2},{a}_{r}={x}_{c}+\frac{w\text{*}\text{r}}{2}$$

16 $$\:{a}_{t}={y}_{c}-\frac{h\text{*}\text{r}}{2},\:{a}_{b}={y}_{c}+\frac{h\text{*}\text{r}}{2}$$

17 $$\:in\_inter=\left({min}\left({b}_{r},{a}_{r}\right)-\text{max}\left({b}_{l},{a}_{l}\right)\right)\text{*}\left({min}\left({b}_{b},{a}_{b}\right)-max({b}_{t},{a}_{t})\right)$$

18 $$\:in\_union=\left({w}^{gt}\text{*}{h}^{gt}\right)\text{*}{\text{r}}^{2}+\left(w\text{*}h\right)\text{*}{\text{r}}^{2}-in\_inter$$

19 $$\:{IoU}^{in}=in\_inter/in\_union$$

The distance loss evaluates the discrepancy in positional alignment between the GT box and its corresponding predicted counterpart along both horizontal and vertical axes, following the computation outlined by Eq. (20). Meanwhile, the shape loss assesses variations in shape size between these two boxes by employing calculations described in Eq. (21).

The variables $\:{w}^{c}$ and $\:{h}^{c}$ represent the width and height of the minimum BD box that covers both the GT box and the PD box, respectively. The coefficient *k* is a weight with a range between 0 and 1. The weights $\:{\text{w}}_{1}$ and $\:{h}_{1}$ correspond to the horizontal and vertical directions, respectively, taking into account the size of the GT box. The scaling factor *s* relates to the target scales in the dataset. Lastly, *c* represents the diagonal distance between boxes *a* and *b*’s minimum enclosing bounding boxes; $\:{\Theta\:}$ denotes attention towards shape loss.

20 $$\:\varDelta\:=\text{k}\text{*}\sum\:_{t=x,y}(1-{e}^{-{\rho\:}_{t}})$$

21 $$\:{\Omega\:}=\frac{1}{2}\sum\:_{t=w,h}{(1-{e}^{-{w}_{t}})}^{{\Theta\:}},{\Theta\:}=\left[\text{2,6}\right]$$

22 $$\:\left\{\begin{array}{c}{\rho\:}_{x}={w}_{1}*{\left(\frac{{x}_{c}-{x}_{c}^{gt}}{{w}^{c}}\right)}^{2}\\\:{\rho\:}_{y}={{h}_{1}\text{*}\left(\frac{{y}_{c}-{y}_{c}^{gt}}{{h}^{c}}\right)}^{2}\end{array}\right.$$

23 $$\:\left\{\begin{array}{c}{\text{w}}_{1}=\frac{{2\text{*}{(w}^{gt})}^{s}}{{{(w}^{gt})}^{s}+{{(h}^{gt})}^{s}}\\\:{h}_{1}=\frac{{2\text{*}{(h}^{gt})}^{s}}{{{(w}^{gt})}^{s}+{{(h}^{gt})}^{s}}\end{array}\right.$$

24 $$\:\left\{\begin{array}{c}{w}_{w}={h}_{1}*\frac{\left|w-\left.{w}^{gt}\right|\right.}{\text{m}\text{a}\text{x}(w,{w}^{gt})}\\\:{w}_{h}={w}_{1}*\frac{\left|h-\left.{h}^{gt}\right|\right.}{\text{m}\text{a}\text{x}(h,{h}^{gt})}\end{array}\right.$$

## Experiments

In the experimental section, we initially present the preparatory work, encompassing relevant information regarding the ACDC dataset, methods for data preprocessing, and details of the experimental environment. Secondly, this article elaborates on the experimental setup for this study, which includes model selection, configurations of training parameters, and metrics employed for model evaluation. Subsequently, the efficacy of the SPPMC module, UECA module, iSD-IoU loss for bounding box regression, and the SA-YOLO model is substantiated through ablation and comparative experiments. Finally, comparative experiments are conducted with additional state-of-the-art OD models to demonstrate the advantages of the SA-YOLO model.

### Experimental preparation

#### Dataset

The cardiac MRI medical image dataset studied in this paper is a publicly available dataset, which comes from the 2017 Automated Cardiac Diagnosis Challenge (ACDC) of the Medical Image Computing and Computer Assisted Intervention Society (MICCAI). The data in this dataset were collected by the University Hospital of Dijon in France over 6 years, comprising 150 patients, each with 28 to 40 image scans. In the ACDC dataset, the patients are categorized into five groups based on pathology: Normal Cardiacs (NOR), previous Myocardial Infarction (MINF), Hypertrophic Cardiomyopathy (HCM), Dilated Cardiomyopathy (DCM), and Abnormal Right Ventricle (ARV).

**Fig. 3:**
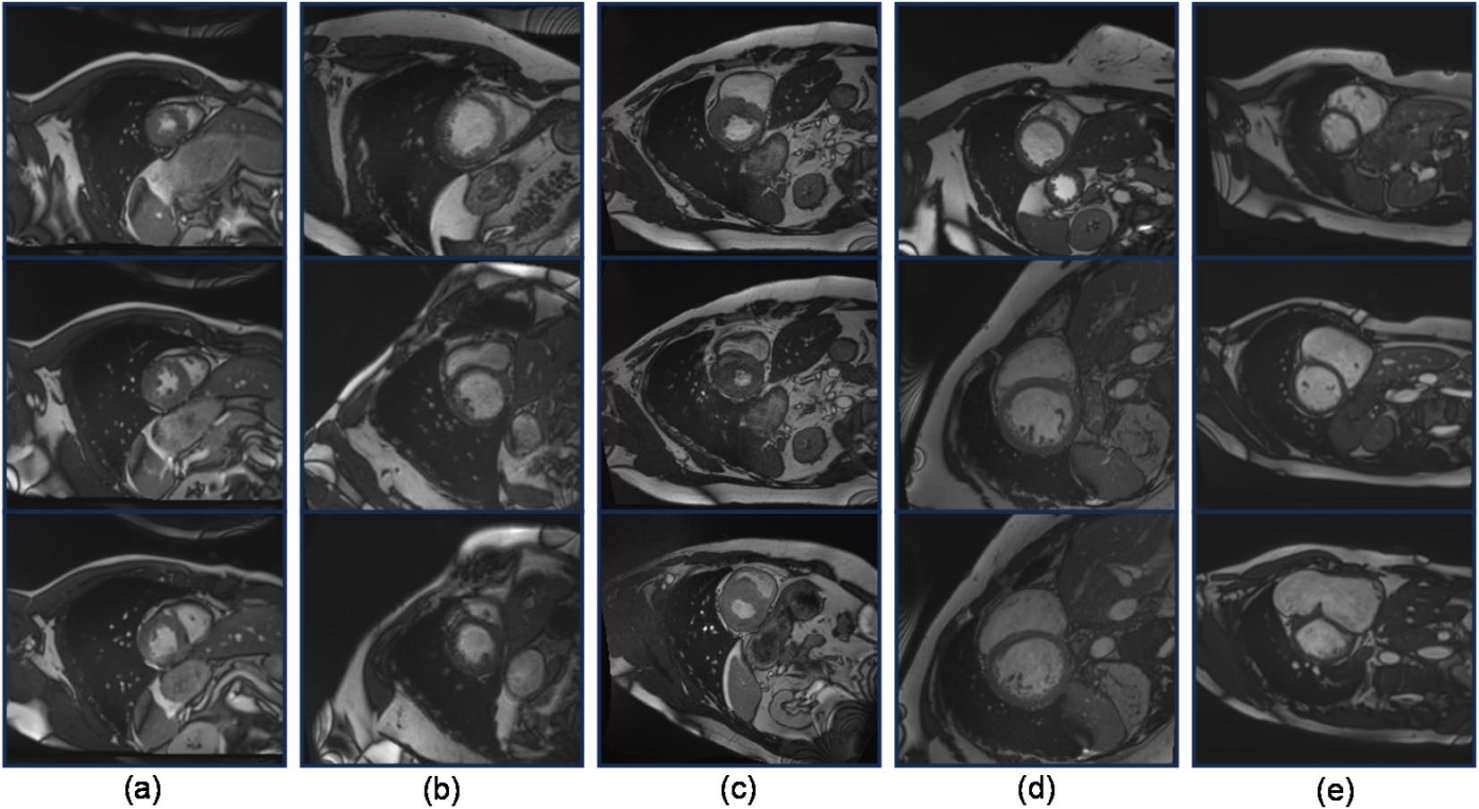
Cardiac MRI medical images in an ACDC dataset. (**a**) Normal Cardiacs; (**b**) Previous Myocardial Infarction; (**c**) Hypertrophic Cardiomyopathy; (**d**) Dilated Cardiomyopathy; (**e**) Abnormal Right Ventricle.

Since the image data used for OD models are typically in formats such as JPEG or PNG, while the images in the ACDC dataset are in the nii.gz format, conversion of the format is required. The converted images need to be annotated according to the standard before they can be used for training OD models. Here, the labeling tool used is LabelImg. Additionally, to minimize interference from the data itself on the model, we conducted a screening process on the acquired image data, excluding images of low quality and those with missing targets. Ultimately, we obtained 2682 images, with a partition ratio of approximately 7:2:1 for the training, testing, and validation sets, respectively. Some image data are shown in Fig. 3.

#### Experimental environment

The experiments in this study were conducted on the same computer equipment to ensure consistency in the experimental environment and reliability of the results. The detailed configuration is provided in Table 1. In terms of hardware, a 13th generation Intel(R) Core (TM) i5-13400 F CPU running at 2.50 GHz was utilized, along with an NVIDIA GeForce RTX 3060Ti GPU. Regarding software, Windows 11 served as the Operating System (OS), Anaconda3 was used as the Integrated Development Environment (IDE), Python 3.9.16 was employed as the programming language, Pytorch 2.0.0 was chosen as the deep learning framework, CUDA 11.2 facilitated accelerated computing, and cuDNN 8.1.1 was employed for deep learning tasks.

**Table 1:** Experimental equipment configuration table.

Configuration	Name	Detailed Information
Hardware	CPU	Intel i5 13,400 F
	GPU	RTX 3060Ti
Software	OS	Windows11
	IDE	Anaconda3
	Python	version:3.9.16
	Pytorch	version:2.0.0
	CUDA	version:11.2
	cuDNN	version:8.1.1

### Experimental detail

#### Models training parameter setting

During the training process, Table 2 presents the key hyperparameter configurations of the SA-YOLO model. The hyperparameters not listed in Table 2 remain unchanged, and other advanced OD models are trained using their default hyperparameters. Moreover, all OD models were trained for 200 epochs with a batch size of 8 and an input image resolution of 640 × 640. A confidence threshold of 0.55 was set for OD during predictions. To ensure objectivity, the final results were averaged over ten experimental runs. No pre-trained weight files were utilized in the training of any models.

**Table 2:** Hyperparameters of the SA-YOLO Model.

Hyperparameter	Value	Hyperparameter	Value
Optimizer	SGD	Batch Size	8
original Learning Rate	0.01	Input Resolution	640 × 640
Weight Decay	0.0005	Epochs	200
Momentum	0.8	conf	0.55

#### Model evaluation

The performance of the OD model in this paper is quantitatively evaluated using Average Precision (AP), mAP0.5, mAP0.5-0.95, F1 score, FPS, and GFLOPs as evaluation metrics. The definitions and computation methods for each metric are presented in Table 3. In this paper, AP is used to represent the AP value when the IoU threshold is 0.5; the mAP0.5 indicates the mean of AP across all classes at an IoU threshold of 0.5; the mAP0.5-0.95 represents the mean of AP across all classes over a range of IoU thresholds from [0.5, 0.95]. True Positive (TP) indicates the number of samples that the model predicts to be positive and are actually positive. False Positive (FP) indicates the number of samples that the model predicts to be positive but are actually negative. False Negative (FN) indicates the number of samples that the model predicts to be negative but are actually positive.

**Table 3:** Evaluation Metric Summary table.

Evaluation Metric	Definition	Computing formula
P	Precision (P) measures the proportion of samples that are actually positive when the model predicts them to be positive.	$\:\text{P}=\frac{TP}{TP+FP}$
R	Recall (R) measures the ability of the model to identify all actual positive samples.	$\:\text{R}=\frac{TP}{TP+FN}$
F1 score	The F1 score is a metric that combines Precision (P) and Recall (R) through a weighted average.	$\:\text{F}1=\frac{2PR}{P+R}$
AP	The AP is a metric utilized to evaluate the performance of OD models by measuring the accuracy of detecting different classes of objects.	The area bounded by the P-R curve corresponds to the value of AP.
mAP	mAP stands for Mean Average Precision.	$\:mAP=\frac{\sum\:_{i=1}^{N}{AP}_{i}}{N}$
FPS	FPS stands for Frames Per Second, a measure of the rate at which consecutive frames or images are displayed or processed.	$\:FPS=\frac{1}{\text{L}\text{a}\text{t}\text{e}\text{n}\text{c}\text{y}}$
GFLOPs	GFLOPs are billions of floating**-**point operations, which can be used to measure the complexity of an algorithm.	The larger the value, the more complex the model.

### Experiment results

#### The ablation experiment

**Fig. 4:**
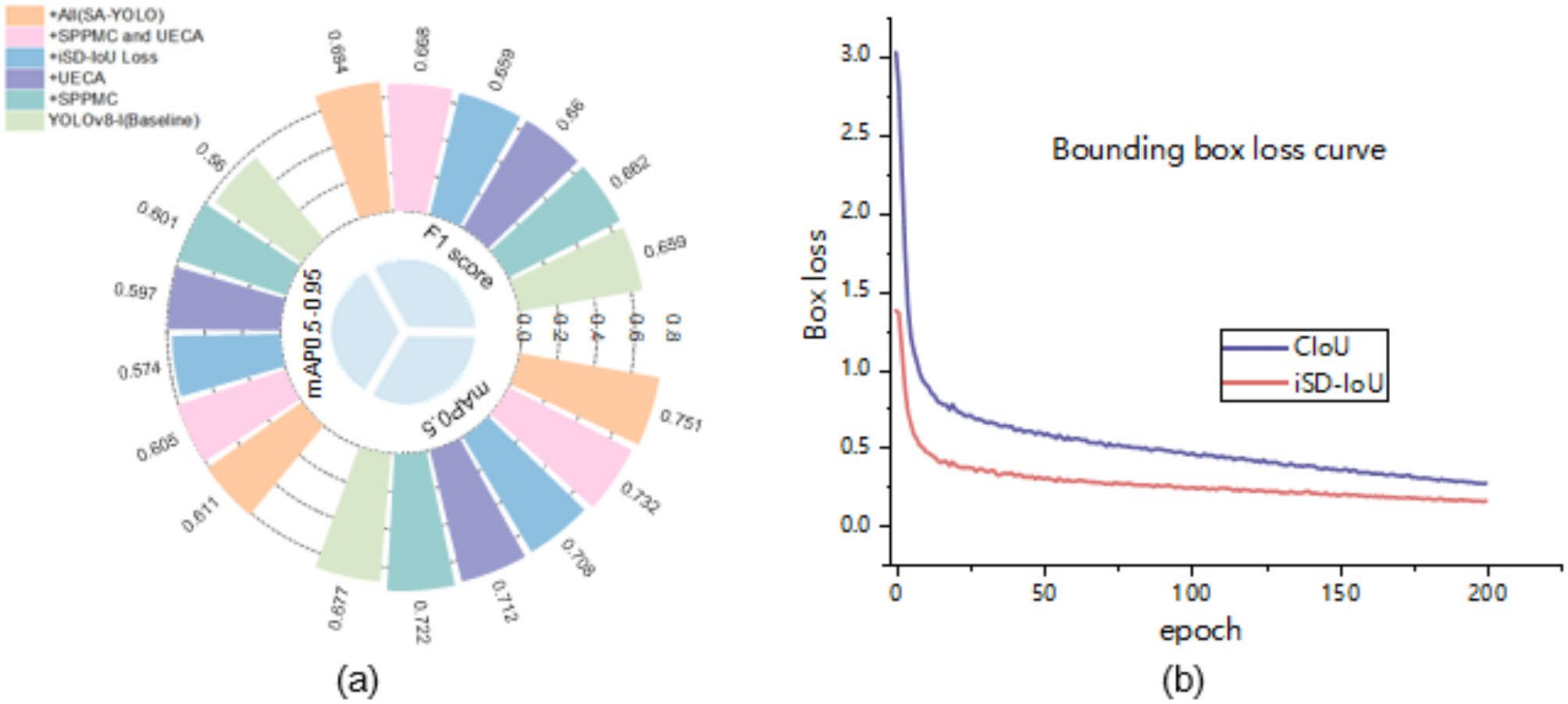
Visualization of the ablation experiment results. (**a**) Radial bar chart of the ablation experiment results; (**b**) Convergence curves of CIoU loss and iSD-IoU loss.

**Fig. 5:**
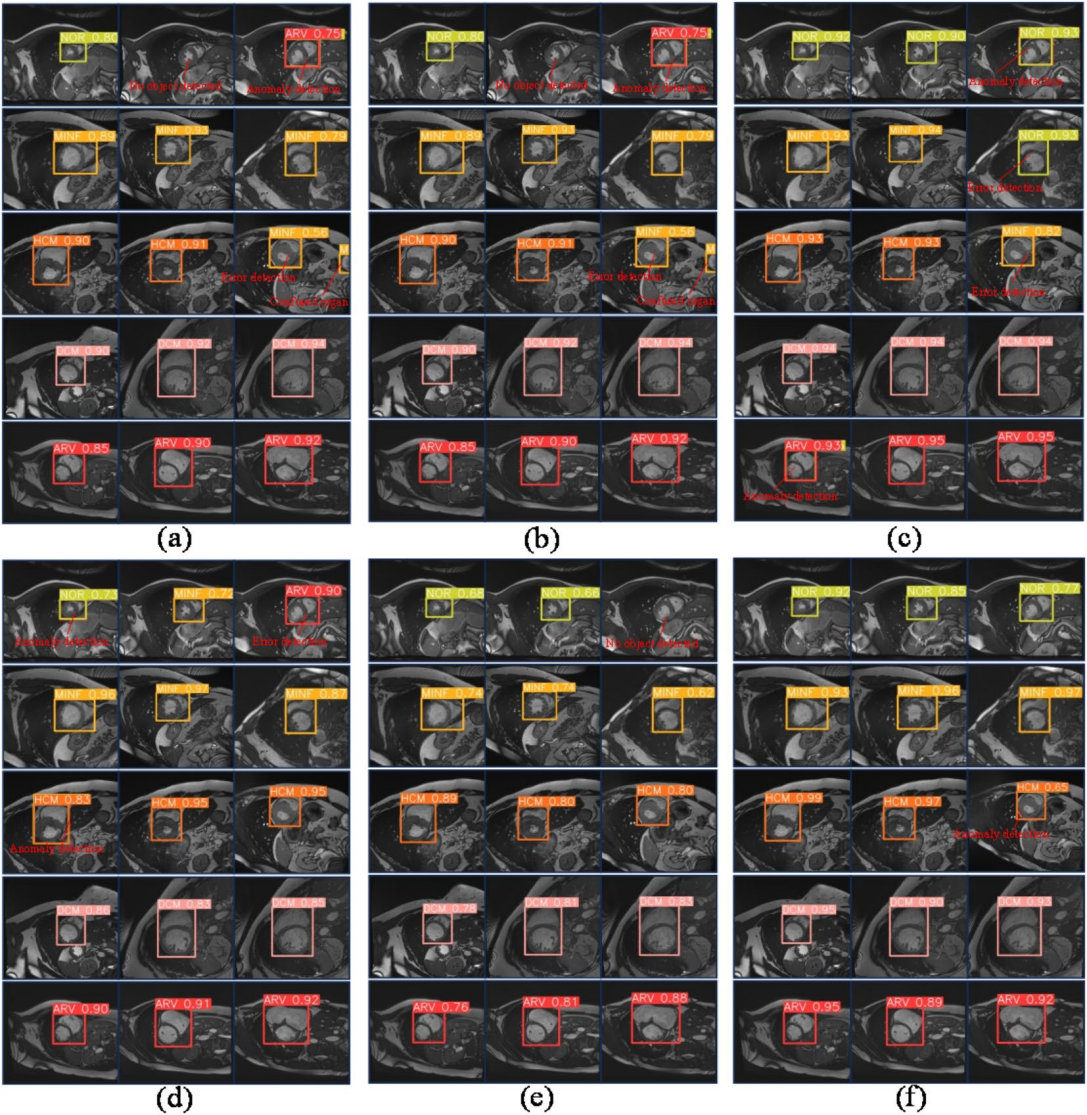
The diagram illustrates the predictive outcomes of the model involved in the ablation experiment. (**a**) Baseline model; (**b**) The baseline model incorporates the SPPMC module. (**c**) The baseline model incorporates the UECA module; (**d**) The baseline model incorporates the iSD loss function; (**e**) The baseline model incorporates the SPPMC module and the UECA module; (**f**) The SA-YOLO model exhibits superior accuracy in predicting cardiac pathologies. (Notes: “No object detected” indicates failure in OD on the image; “Anomaly detection” suggests the model predicts multiple categories including itself within a single bounding box; “Error detection” denotes misclassification by the model; “Confused organ” signifies misidentification of other organs as the heart.)

The YOLOv8-l model is employed as the baseline in this study, and a series of ablation experiments are conducted on the ACDC dataset to validate the efficacy of each proposed enhancement. The results obtained from these ablation experiments can be found in Tables 4 and 5, and 6; Figs. 4 and 5. Discussion on the selection of maximum pooling layer kernel size for the SPPMC module as Table 4, and in the SPPMC module, a group using the size parameters of the pooling kernel designed by this article has achieved the best experimental results, which proves the effectiveness of the design strategy.

**Table 4:** Discussion on the selection of maximum pooling layer kernel size for the SPPMC module.

maximum pooling kernel sizes	F1	mAP 0.5	mAP 0.5–0.95	GFLOPs
3,5,7,9	0.592	0.689	0.584	175.4
5,7,9,11	0.627	0.671	0.571	175.4
7,9,11,13	0.638	0.675	0.561	175.4
9,11,13,15	0.637	0.693	0.577	175.4
11,13,15,17	0.642	0.704	0.589	175.4
5,9,13,17(Ours)	**0.662**	**0.722**	**0.601**	175.4

**Table 5:** Loss function ablation experiment.

BBR Loss Function Classes	F1	mAP 0.5	mAP 0.5–0.95	GFLOPs
DIoU	0.630	0.689	0.579	167.5
CIoU	**0.659**	0.677	0.560	167.5
SIoU	0.656	0.702	**0.594**	167.5
Shape-IoU	0.655	0.689	0.569	167.5
Inner-IoU	0.658	0.695	0.572	167.5
iSD-IoU (Ours)	**0.659**	**0.708**	0.574	167.5

**Table 6:** Ablation experiment results Summary table.

YOLOv8-l	SPPMC	UECA	iSD-IoU	F1	mAP 0.5	mAP 0.5–0.95	FPS	GFLOPs
✓				0.659	0.677	0.560	65.12	167.5
✓	✓			0.662	0.722	0.601	61.91	175.4
✓		✓		0.660	0.712	0.597	**65.37**	169.7
✓			✓	0.659	0.708	0.574	61.65	167.5
✓	✓	✓		0.668	0.732	0.605	61.48	**175.6**
✓	✓	✓	✓	**0.684**	**0.751**	**0.611**	61.21	**175.6**

As shown in Table 6; Fig. 4(a), replacing the SPPF module with the SPPMC module in the baseline model effectively expands the neural network’s receptive field and employs a unique information fusion strategy, thereby enabling more efficient processing of acquired feature information and resulting in a significant enhancement in performance. In comparison to the baseline model, although there is a slight decrease in detection speed, and the computational complexity has increased, incorporating the SPPMC module leads to an increase of 0.3% in the F1 score, along with improvements of 4.5% and 4.1% for mAP0.5 and mAP0.5-0.95 respectively, demonstrating convincingly its efficacy for enhancing model performance.

After incorporating the UECA module independently into the baseline model, the network’s ability to capture contextual information and expand its perceptive range is enhanced, while attention to key feature information is heightened. This enables more effective processing of acquired feature information, resulting in a significant performance improvement. Compared to the baseline model, introducing the UECA module leads to an increase in detection speed with the F1 score rising by 0.1%, and mAP0.5 and mAP0.5-0.95 improving by 3.5% and 3.7%, respectively. These results confirm that integrating the UECA module effectively enhances model performance.

Subsequently, after independently substituting the original CIoU loss with the enhanced BBR loss function iSD-IoU loss in the baseline model, a significant enhancement in model performance is observed. While experiencing a slight reduction in detection speed, there is no shift in the F1 score; however, mAP0.5 and mAP0.5-0.95 increase by 3.1% and 1.4%, respectively. Moreover, In the ablation experiments of various BBR loss functions shown in Table 5, the performance of the target measurement model has been well improved after the addition of the loss function without sacrificing the complexity of the model. The data of the other evaluation indicators rank the first place, except for the index mAP 0.5–0.95, which is slightly lower. Figure 4(b) demonstrates that when utilizing the iSD-IoU loss function, convergence occurs at a faster rate with lower loss values for the bounding box loss functions employed. In summary, the strategy of using the iSD-IoU loss function helps to speed up the model convergence and improve the overall performance of the model.

By incorporating the SPPMC and UECA modules into the baseline model as per the strategy outlined in Fig. 1, there is a significant enhancement in model performance. The F1 score experiences an increase of 0.9%, while mAP0.5 and mAP0.5-0.95 see respective increases of 5.5% and 4.5%. Building upon this improvement, replacing the CIoU loss function with the iSD-IoU loss function leads to a significant enhancement in performance within the SA-YOLO model. In comparison to the baseline network model, although the detection speed is reduced, and the computational complexity is increased, but there is an observed increase of 2.5% in the F1 score and improvements of 7.4% and 5.1% in mAP0.5 and mAP0.5-0.95, respectively. Furthermore, 15 random images from the MRI medical images of the heart that were not included in the model training and validation process were selected for each category, with three images per category, to investigate the detection and recognition capabilities of various cardiovascular pathologies by the models. The results are presented in Fig. 5. The baseline model exhibits susceptibility to misclassification, failure in OD, anomaly detection, and confusion organs. With the incorporation of the improved module in this study, these problems were effectively mitigated, and the model was able to better resist interference from complex backgrounds and more accurately discriminate between various types of complex cardiac lesions. Importantly, in all ablation experiments, it is easy to see that the SA-YOLO model is able to predict different cardiac lesions more accurately and with higher confidence than the baseline model, and also demonstrates excellent discrimination of cardiac lesions with smaller pixels. This further confirms the advance and validity of our proposed SA-YOLO model.

#### The comparative experiment of spatial pyramid pooling modules

**Fig. 6:**
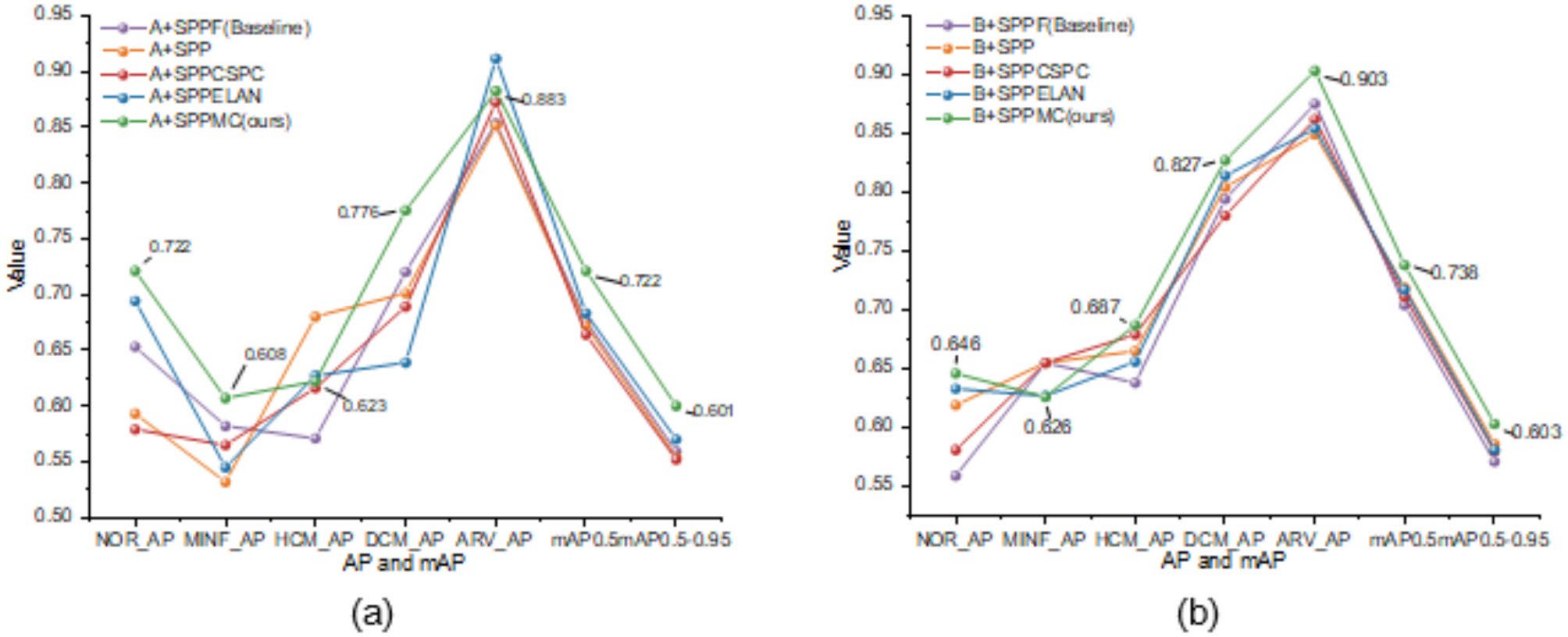
The experimental results of the model on the ACDC dataset are visualized through the integration of diverse spatial pyramid pooling modules. (**a**) Model A adds the different spatial pyramid pooling modules; (**b**) Model B adds the different spatial pyramid pooling modules.

**Fig. 7:**
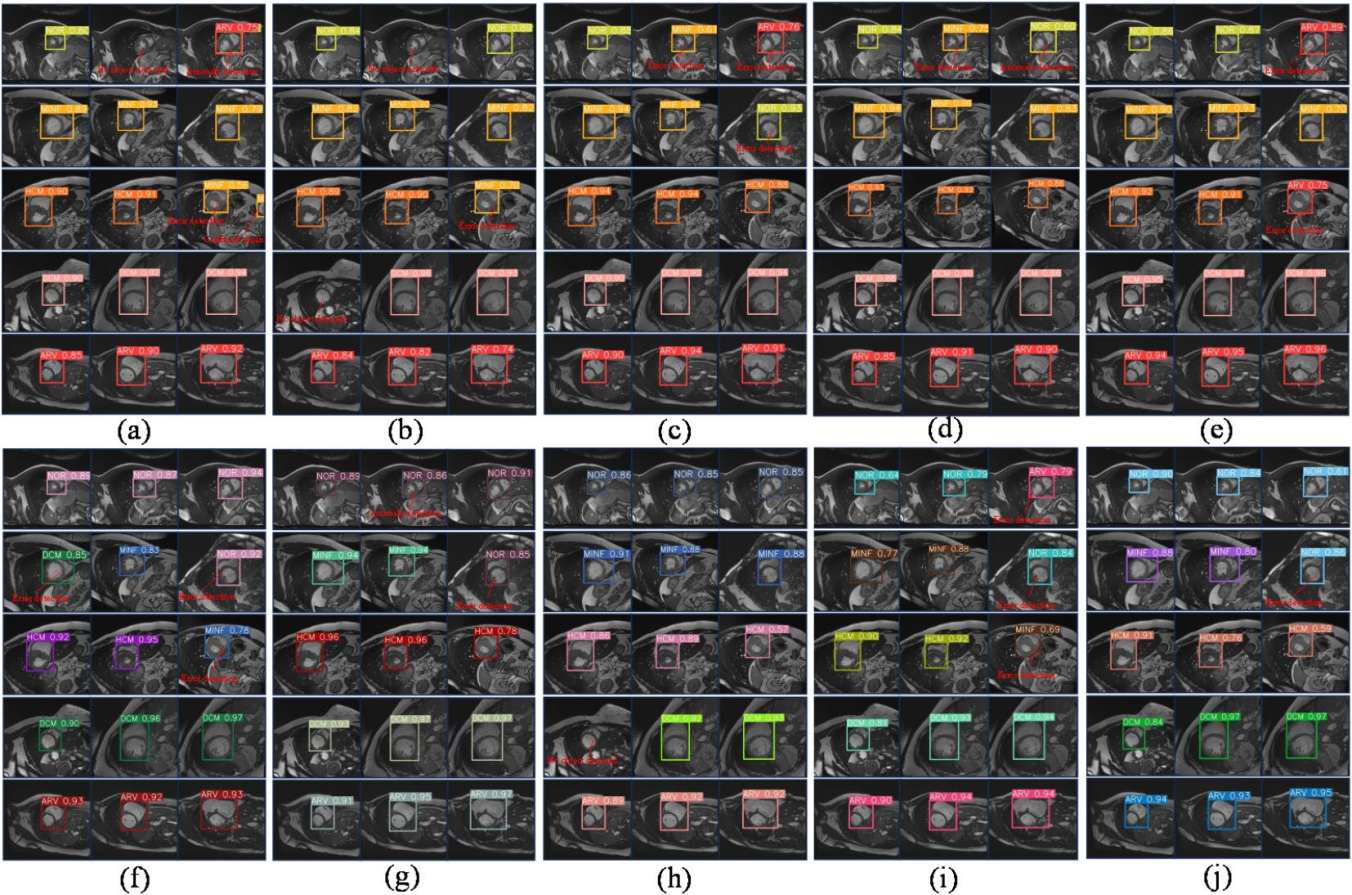
The prediction outcomes of cardiac pathology in model A and model B following the incorporation of diverse spatial pyramid pooling modules. (**a**) Model A adds the SPPF module; (**b**) Model A adds the SPP module; (**c**) Model A adds the SPPCSPC module; (**d**) Model A adds the SPPELAN module; (**e**) Model A adds the SPPMC module; (**f**) Model B adds the SPPF module; (**g**) Model B adds the SPP module; (**h**) Model B adds the SPPCSPC module; (**i**) Model B adds the SPPELAN module; (**j**) Model B adds the SPPMC module.

To further demonstrate the advantages of the SPPMC module, this study integrates the SPPMC module, with the baseline models YOLOv8-l (denoted as A) and YOLOv7^39^ (denoted as B) and conducts comparative experiments with other spatial pyramid pooling networks. The other spatial pyramid pooling modules tested include the SPP^30^, SPPF, SPPCSPC, and SPPELAN. The experimental implementation details remained unaltered. The experiments and visual representations are presented in Table 7; Figs. 6 and 7.

**Table 7:** After adding various spatial pyramid Pooling Network modules, the model’s experimental results on the ACDC dataset.

Method	AP					mAP0.5	mAP 0.5–0.95
	NOR	MINF	HCM	DCM	ARV		
A + SPPF(Baseline)	0.654	0.583	0.572	0.721	0.854	0.677	0.560
A + SPP	0.594	0.533	**0.681**	0.702	0.852	0.673	0.555
A + SPPCSPC	0.580	0.566	0.617	0.690	0.873	0.665	0.553
A + SPPELAN	0.695	0.546	0.628	0.640	**0.912**	0.684	0.571
A + SPPMC(Ours)	**0.722**	**0.608**	0.623	**0.776**	0.883	**0.722**	**0.601**
B + SPPF(Baseline)	0.559	**0.655**	0.638	0.794	0.875	0.704	0.571
B + SPP	0.619	**0.655**	0.665	0.804	0.849	0.719	0.586
B + SPPCSPC	0.581	**0.655**	0.679	0.780	0.862	0.711	0.580
B + SPPELAN	0.633	0.627	0.656	0.814	0.854	0.717	0.581
B + SPPMC(Ours)	**0.646**	0.626	**0.687**	**0.827**	**0.903**	**0.738**	**0.603**

The performance of models A and B exhibited significant improvement after the incorporation of the SPPMC module, as evident from Table 7; Fig. 6(a), and Fig. 6(b), leading to superior detection results. After incorporating the SPPMC module in model A, there is a small decrease in the AP values for the two categories, yet surpasses that of baseline models, while the remaining categories exhibit the highest AP values. Furthermore, both mAP0.5 and mAP0.5-0.95 values significantly exceed those of other models, with improvements of 4.5% and 4.1%, respectively, compared to the baseline network. After incorporating the SPPMC module in model B, there is a slight decrease in the AP values for one category, while the remaining categories exhibit the highest AP values. The mAP0.5 and mAP0.5-0.95 values exhibit higher than the other models, showcasing respective increments of 3.4% and 3.2% compared to the baseline network.

The replacement of the spatial pyramid pooling modules in model A and model B significantly improved the prediction efficacy for cardiac pathology. After conducting a comprehensive comparative analysis, it was observed that the model incorporating the SPPMC module exhibited superior predictive performance for cardiac pathology. The integration of the SPPMC module into model A, as depicted in Fig. 7(a) and Fig. 7(e), resulted in a significant reduction in instances of missed detections, detection anomalies, and organ misclassifications, and it led to an improvement in the confidence of detection results for the images. In addition, Fig. 7(f) and Fig. 7(j) demonstrate that the performance of model B was significantly enhanced with the incorporation of the SPPMC module. This enhancement resulted in a reduction of erroneous predictions and an increase in the confidence level of detection results for the images. Based on these findings, this study concludes that the SPPMC module effectively enhances OD models’ performance in cardiac pathology tasks, surpassing classical spatial pyramid pooling modules’ achievements.

#### Comparative experiments on improved attention mechanism networks

**Fig. 8:**
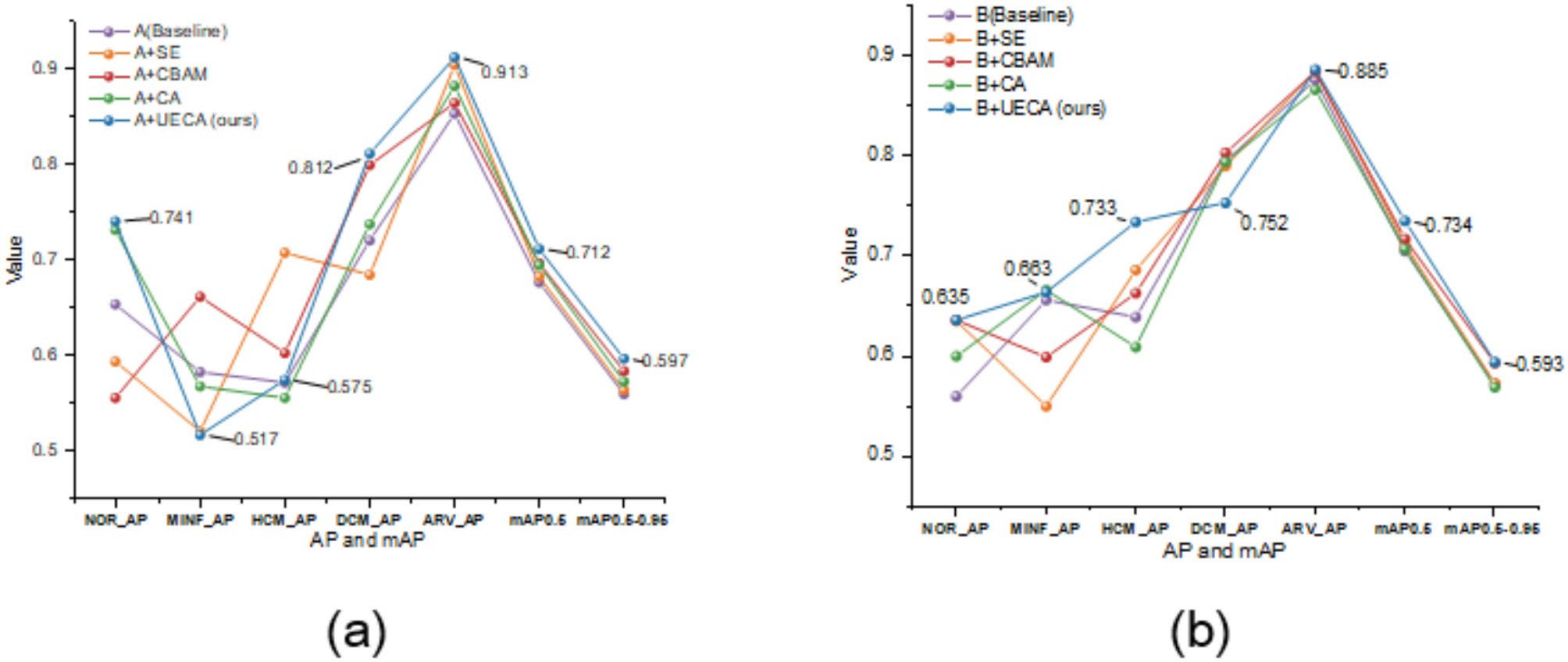
The experimental results of the model on the ACDC dataset are visualized through the integration of diverse attention mechanism modules. (**a**) Model A adds the various modules for attention mechanisms; (**b**) Model B adds the various modules for attention mechanisms.

**Fig. 9:**
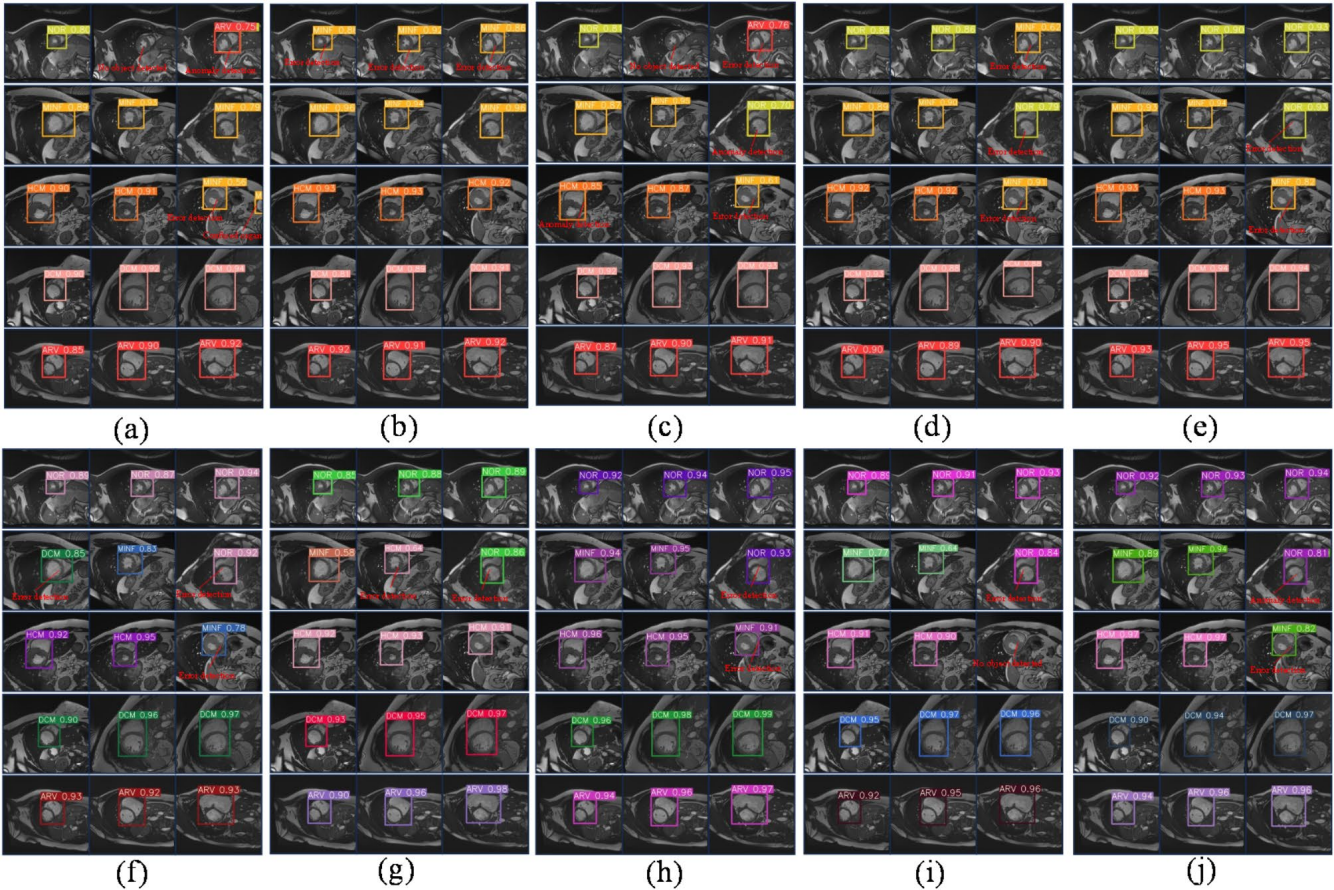
The prediction outcomes of cardiac pathology in model A and model B following the incorporation of diverse attention mechanism modules; (**a**) Model A, (**b**) Model A adds the SE module; (**c**) Model A adds the CBAM module; (**d**) Model A adds the CA module; (**e**) Model A adds the UECA module; (**f**) Model B; (**g**) Model B adds the SE module; (**h**) Model B adds the CBAM module; (**i**) Model B adds the CA module; (**j**) Model B adds the UECA module.

To further demonstrate the UECA module’s effectiveness, we incorporate the UECA attention mechanism module and other attention mechanism modules (for example: SE, CA, and CBAM^31^) into the network architectures of baseline models A and B for comparative experiments. No modifications are made to the experimental implementation details. The experimental results and visualizations are presented in Table 8; Figs. 8 and 9.

**Table 8:** After adding different types of attention mechanism modules, the experimental results of the model on the ACDC dataset.

Method	AP					mAP0.5	mAP 0.5–0.95
	NOR	MINF	HCM	DCM	ARV		
A(Baseline)	0.654	0.583	0.572	0.721	0.854	0.677	0.560
A + SE	0.594	0.521	**0.708**	0.685	0.905	0.683	0.564
A + CBAM	0.556	**0.662**	0.603	0.800	0.865	0.697	0.584
A + CA	0.732	0.568	0.556	0.738	0.883	0.695	0.573
A + UECA (Ours)	**0.741**	0.517	0.575	**0.812**	**0.913**	**0.712**	**0.597**
B(Baseline)	0.559	**0.655**	0.638	0.794	0.875	0.704	0.571
B + SE	0.634	0.549	0.685	0.789	0.884	0.710	0.572
B + CBAM	**0.635**	0.598	0.662	**0.802**	0.883	0.716	0.592
B + CA	0.599	**0.665**	0.608	0.793	0.865	0.706	0.568
B + UECA (Ours)	**0.635**	0.663	**0.733**	0.752	**0.885**	**0.734**	**0.593**

From Table 8; Fig. 8(a), and Fig. 8(b), it is evident that the performance of models A and B significantly improved after adding the UECA module. In model A, after incorporating the UECA network, the AP value for two categories is higher than all other models, and for one category, it is lower than all other models; the AP values for the remaining categories are higher than those of most models. Both mAP0.5 and mAP0.5-0.95 values are higher than those of all other models, with increases of 3.5% and 3.7%, respectively, compared to the baseline network. In model B, after adding the UECA network, three categories’ AP values are the highest among all models, and one category’s AP value is the lowest; the AP values for the remaining categories are slightly lower than those of a few models. Both mAP0.5 and mAP0.5-0.95 values are higher than those of all other models, with increases of 3.0% and 2.2%, respectively, compared to the baseline network.

The replacement of the attention mechanism modules in model A and model B resulted in a significant improvement in the prediction efficacy for cardiac pathology. After conducting a comprehensive comparative analysis, it was observed that the model incorporating the UECA module exhibited superior predictive performance for cardiac pathology. From Fig. 9(a) and Fig. 9(e), it can be seen that when the UECA module is added to model A, although the number of error detection images increases by one image, the omission of detection, prediction abnormality, and organ confusion are effectively improved, and the number of images with improvement in the confidence of prediction results increased. Figure 9(f) and Fig. 9(j) reveal that upon adding the UECA module to model B, there is a reduction in detection errors, and more images exhibit increased confidence in the predicted results. Taking all factors into consideration, this study concludes that the UECA module effectively enhances the performance of the model in cardiac pathology detection, demonstrating superiority over the aforementioned classic attention mechanism modules.

#### Performance comparison experiment of the OD model

**Fig. 10:**
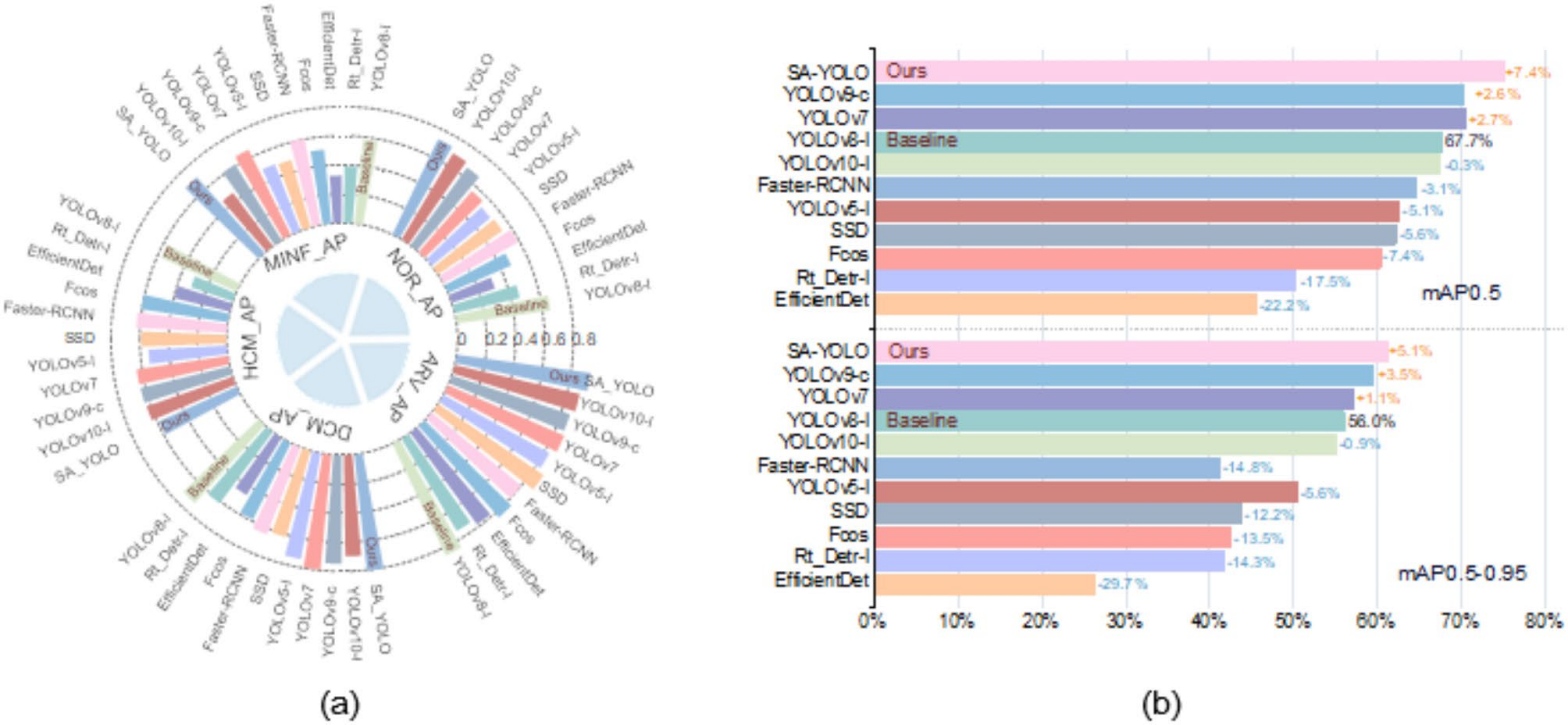
Visualization of the comparative experimental results of OD models on the ACDC dataset. (**a**) Radial bar chart showing AP values for different cardiac pathologies, and (**b**) Bar chart showing mAP0.5 and mAP0.5-0.95 values.

In this study, we compared our proposed SA-YOLO algorithm with several cutting-edge OD algorithms, including Rt-Detr^40^, EfficientDet^41^, Fcos^42^, Faster-RCNN, SSD, YOLOv5-l^43^, YOLOv7, YOLOv8-l, YOLOv9-c^44^, and YOLOv10-l^45^. The objective is to demonstrate the strong competitiveness of our model in detecting cardiac MRI medical images. We conducted comparative experiments on the ACDC dataset using these aforementioned OD models. The experimental results are summarized in Table 9; Fig. 10.

Through the analysis of Table 9; Fig. 10, it can be observed that, locally, the SA-YOLO model demonstrates superior performance in detecting Normal Cardiacs, previous Myocardial Infarction, Dilated Cardiomyopathy, and Hypertrophic Cardiomyopathy, with AP scores higher than other models. Additionally, in the detection and recognition of Abnormal Right Ventricle, the SA-YOLO model also ranks among the top performers, with AP scores still higher than most other advanced OD models, including baseline models, albeit slightly lower than individual advanced OD models. Furthermore, from the numerical values of the AP scores, it is evident that most models exhibit the best detection performance for Dilated Cardiomyopathy and Abnormal Right Ventricle, while the detection performance for Normal Cardiacs, previous Myocardial Infarction, and Hypertrophic Cardiomyopathy is slightly inferior. We attribute these results to the following reasons:

1. The MRI medical image data for various cardiac pathologies are not evenly balanced, and there is a certain lack of data volume.
2. Due to the variability among individual human hearts, some features of MRI images for certain cardiac pathologies may exhibit similarities, and interference from other tissue organs may lead to misjudgments in OD models.

**Table 9:** OD model comparison experimental results summary table.

Method	AP					mAP 0.5	mAP 0.5–0.95
	NOR	MINF	HCM	DCM	ARV		
YOLOv8-l(Baseline)	0.654	0.583	0.572	0.721	0.854	0.677	0.560
Rt_Detr-l	0.461	0.389	0.305	0.613	0.741	0.502	0.417
EfficientDet	0.313	0.325	0.394	0.462	0.782	0.455	0.263
Fcos	0.474	0.510	0.605	0.587	0.839	0.603	0.425
Faster-RCNN	0.586	0.606	0.625	0.642	0.800	0.646	0.412
SSD	0.535	0.482	0.592	0.617	0.880	0.621	0.438
YOLOv5-l	0.523	0.490	0.542	0.742	0.835	0.626	0.504
YOLOv7	0.559	0.655	**0.638**	0.794	0.875	0.704	0.571
YOLOv9-c	0.664	0.611	0.635	0.747	0.860	0.703	0.595
YOLOv10-l	0.692	0.460	0.630	0.697	0.888	0.674	0.551
SA-YOLO(Ours)	**0.728**	**0.700**	0.587	**0.805**	**0.933**	**0.751**	**0.611**

Overall, the SA-YOLO model demonstrates exceptional performance on the ACDC dataset compared to other state-of-the-art models, securing first place in accurately detecting cardiac pathologies in MRI medical images. The mAP0.5 and mAP0.5-0.95 scores stand at 0.751 and 0.611, respectively, significantly surpassing the performance of other advanced OD models. Additionally, the mAP0.5 and mAP0.5-0.95 scores have witnessed a significant increase of 7.4% and 5.1%, respectively, when compared to the baseline model YOLOv8-l. In comparison to the second-ranked YOLOv9-c model, there has been an improvement of 4.8% and 1.6% in the mAP0.5 and mAP0.5-0.95 scores, respectively. Furthermore, when contrasted with the last-ranked EfficientDet model, remarkable enhancements of 29.6% and 34.8% have been achieved in the mAP0.5 and mAP0.5-0.95 scores.

In summary, SA-YOLO exhibits exceptional performance in the detection of cardiac pathologies in cardiac MRI medical images compared to other advanced OD models. It offers significant improvements in performance and strong competitiveness.

### Discussion

This paper addresses the challenges in detecting cardiac MRI images in medical imaging and proposes an enhanced OD algorithm model, called SA-YOLO. A considerable number of experimental results on the ACDC dataset demonstrate that the improved strategy proposed in this paper can effectively improve the accuracy and reliability of the target detection model for cardiac pathology detection. Performance can be improved by adding a single improvement module or all of them to the baseline model. With the addition of a single module, the baseline model’s mAP0.5 and mAP0.5-0.95 scores mostly rose increased by more than 2%. When all the improved modules were added to the baseline model, the F1, mAP0.5 and mAP0.5-0.95 scores increased significantly, to 2.5%, 7.4%, and 5.1%, respectively. Compared with other advanced object detection models, the SA-YOLO model performs well, ranking first with mAP0.5 and mAP0.5-0.95 scores.

There are still some limitations in this study. First, there is still a lot of room for improvement in the performance of the model. When the SPPMC or UECA module is introduced into the baseline model, the complexity of the model will increase to a certain extent. In addition, although 2682 images are a good starting point for an object detection task, in the medical field, the total amount of the data set is still slightly insufficient, which may cause the model to perform poorly on new and unseen samples. This dataset has achieved good performance in the current object detection task, and the clinical application of the model also faces some challenges. Before clinical use, rigorous verification and evaluation are still required.

## Conclusion

This paper conducts in-depth research on multi-scale feature extraction and fusion, training process optimization, and Positioning accuracy improvement of the target detection model, intending to solve the problems of low detection accuracy and unreliable detection results of target detection models for cardiac pathology detection on cardiac MRI medical images. Based on the YOLOv8 model, this paper proposes a cardiac pathology detection model with cardiac MRI medical images, which introduces the innovative SPPMC module, UECA module, and iSD-IoU loss function. the SPPMC module solves the limitations of the traditional spatial pyramid pooling network, enhances the flexibility of the network to handle multi-scale feature maps, and helps the backbone network to extract deeper and richer multi-scale features, etc. The UECA module considers the effects of channel attention and spatial attention at the same time and inherits the advantages of CA and SE. The network can better adjust the feature weights dynamically according to the input feature maps, inhibit the perturbation of non-essential features, and enhance the attention of important features, which effectively promotes feature fusion in the neck network. iSD-IoU loss function is designed to optimize the training process of the model further and to enhance the detection performance of the model, which introduces an auxiliary box to improve the detection performance, which introduces the idea of an auxiliary frame to calculate the IoU and considers the effects of shape loss and distance loss at the same time. A large number of experiments on the ACDC dataset verify the effectiveness of the improved strategy in this paper, and the SA-YOLO model, with a small amount of computational complexity sacrificed, significantly improves its performance, and performs well in the detection task of cardiac pathology, obtaining more reliable and better detection results than other advanced target detection models。.

In the future, the authors’ team will combine the concept of more advanced target detection models to continue to improve the performance of the SA-YOLO model in cardiac MRI medical image detection tasks, while reducing the complexity of the model and easing the difficulty of model deployment. Secondly, the author team will continue to carry out in-depth cooperation with partner hospitals to address the limitations of the ACDC dataset, gradually increase the number of data in the study, make the data more representative, and solve the challenges of clinical application of the model.

## Data Availability

The ACDC dataset used in this paper is a publicly available dataset, and the relevant data on which the results of this paper are based can be obtained from the corresponding author upon reasonable request.
